# The zinc finger protein Zn72D and DEAD box helicase Belle interact and control *maleless *mRNA and protein levels

**DOI:** 10.1186/1471-2199-10-33

**Published:** 2009-04-22

**Authors:** Kathleen A Worringer, Feixia Chu, Barbara Panning

**Affiliations:** 1 Department of Biochemistry and Biophysics, University of California, San Francisco, San Francisco, CA 94158, USA; 2 Gladstone Institute of Cardiovascular Disease, San Francisco, CA 94158, USA; 3 Molecular, Cellular & Biomedical Sciences, University of New Hampshire, Durham, NH 03824, USA

## Abstract

**Background:**

The Male Specific Lethal (MSL) complex is enriched on the single X chromosome in male *Drosophila *cells and functions to upregulate X-linked gene expression and equalize X-linked gene dosage with XX females. The zinc finger protein Zn72D is required for productive splicing of the *maleless *(*mle*) transcript, which encodes an essential subunit of the MSL complex. In the absence of Zn72D, MLE levels are decreased, and as a result, the MSL complex no longer localizes to the X chromosome and dosage compensation is disrupted. To understand the molecular basis of Zn72D function, we identified proteins that interact with Zn72D.

**Results:**

Among several proteins that associate with Zn72D, we found the DEAD box helicase Belle (Bel). Simultaneous knockdown of *Zn72D *and *bel *restored MSL complex localization to the X chromosome and dosage compensation. MLE protein was restored to 70% of wild-type levels, although the level of productively spliced *mle *transcript was still four-fold lower than in wild-type cells. The increase in production of MLE protein relative to the amount of correctly spliced *mle *mRNA could not be attributed to an alteration in MLE stability.

**Conclusion:**

These data indicate that Zn72D and Bel work together to control *mle *splicing and protein levels. Thus Zn72D and Bel may be factors that coordinate splicing and translational regulation.

## Background

There is increasing evidence linking the processes involved in gene expression, such as transcription, splicing, mRNA localization, and translation. For example, splicing factors, such as hnRNP proteins and the serine/arginine-rich (SR) protein SF2/ASF, can remain associated with mature transcripts, shuttle to the cytoplasm, and regulate translation [1-4]. For the *Drosophila oskar *transcript, splicing of the first intron is required for proper localization of the transcript to the posterior end of the oocyte, where it is locally translated [5]. Correct localization of this transcript also depends on the exon junction complex components Y14 and Mago nashi [6-8]. Despite evidence for coordination of splicing, mRNA localization, and translation, the mechanisms underlying this coordination remain poorly characterized.

We previously demonstrated that the *Drosophila *zinc finger protein Zn72D is required for proper splicing of the *maleless *(*mle*) transcript. The MLE protein is an essential component of the Male Specific Lethal (MSL) complex (also known as the Dosage Compensation Complex). The MSL complex is enriched on the sole X chromosome in male cells, where it upregulates gene expression two-fold to equalize gene dosage between XY males and XX females [9]. Zn72D is necessary for productive splicing of *mle *mRNA. Without Zn72D, the majority of *mle *transcripts preferentially retain part of the second intron, which contains in-frame stop codons, and therefore MLE protein is not produced at normal levels [10]. As a result, the MSL complex does not localize to the X chromosome and dosage compensation does not occur.

To further explore the role of Zn72D, we used mass spectrometry to identify proteins that interact with HA-tagged Zn72D. The majority of proteins that co-immunoprecipitate (co-IP) with Zn72D are involved in some aspect of RNA metabolism. One Zn72D-associated protein is the DEAD box helicase Bel, which is implicated in translational regulation [11]. Since Bel associated with Zn72D, we asked whether Bel played a role in regulating *mle *gene expression. Depletion of *bel *alone did not significantly affect the level of MLE or localization of the MSL complex. However, simultaneous knockdown of *Zn72D *and *bel *rescued X chromosome localization of the MSL complex. The level of MLE protein was restored to ~70% of wild-type in the double knockdown, even though the level of productively spliced *mle *transcripts was still four-fold lower than in wild-type cells. These data indicate that Zn72D and Bel work together to control *mle *splicing and protein levels and suggest that Zn72D and Bel may target spliced mRNAs for localized, regulated translation in the cytoplasm.

## Results and discussion

### Identification of proteins that interact with Zn72D

**Figure 1:**
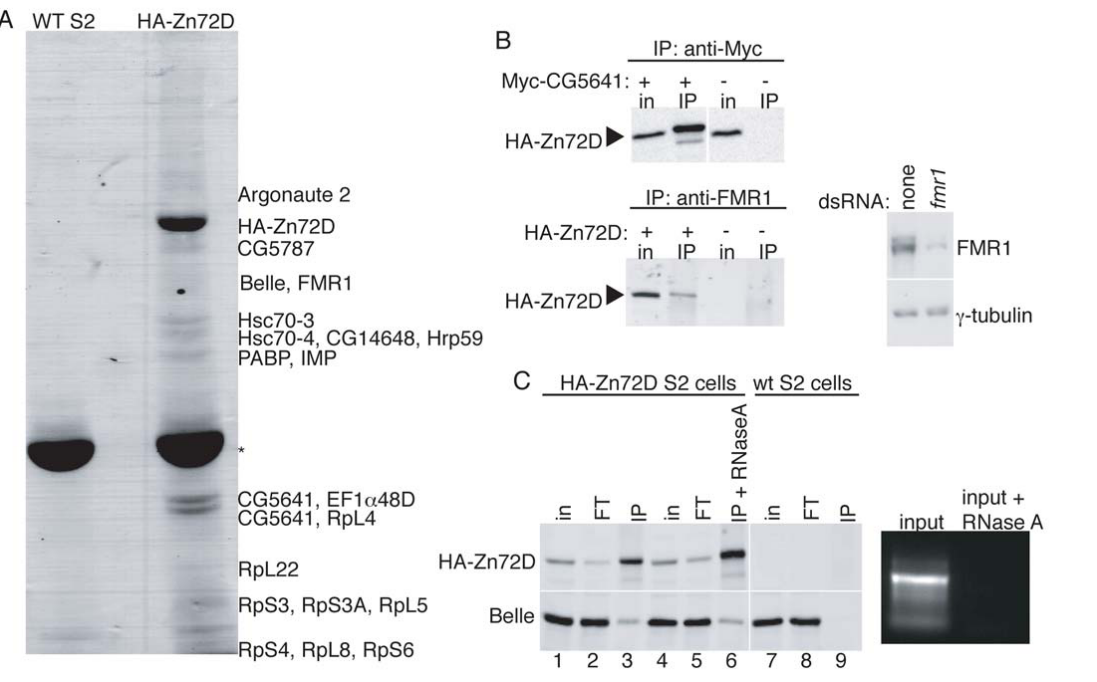
**Identification of proteins that co-immunoprecipitate with Zn72D**. (A) IPs were performed with an anti-HA antibody in wild-type (wt) S2 cells and in S2 cells expressing HA-Zn72D. Proteins that co-IP with HA-Zn72D were identified by mass spectrometry. (B) (Top) HA-Zn72D co-IPs with Myc-CG5641 when cells expressing HA-Zn72D were transiently transfected with a vector expressing Myc-CG5641 (left 2 lanes). IPs were performed using an anti-Myc antibody, and immunoblotting was performed with an HA antibody. Right 2 lanes are controls in which no Myc-CG5641 was transfected. (Bottom left) HA-Zn72D co-IPs with FMR1. IPs were performed using an anti-FMR1 antibody and immunoblotting was performed to detect HA. IPs were performed in cells that did (2 left lanes) and did not (2 right lanes) express HA-Zn72D. (Bottom right) The FMR1 antibody specifically recognizes FMR1 protein. S2 cell extracts from untreated and FMR1 knockdown cells were probed with anti-FMR1 and anti-γ-tubulin. (C) The interaction between Zn72D and Bel is RNA-independent. IPs using anti-HA antibody were performed on S2 cells expressing HA-Zn72D, in the absence (lane 3) or presence (lane 6) of RNase A. Lane 9 is a control IP performed with an anti-HA antibody using S2 cells not expressing HA-Zn72D. HA and Bel were detected using immunoblotting. The ethidium bromide stained gel to the right shows equivalent amounts of input extract loaded per lane, either untreated or treated with RNase A. For parts B and C, "in" indicates 2% of the input into each IP and "FT" indicates 2% of flow-through from each IP.

To gain additional insight into the molecular functions of Zn72D, we carried out tandem mass spectrometric analysis to identify proteins that physically interact with HA-tagged Zn72D in *Drosophila *S2 cells. Proteins that co-IP with anti-HA antibody in S2 cells expressing HA-Zn72D but not in wild-type S2 cells include: Bel, Elongation factor 1α48D (EF1α48D), Fragile × Mental Retardation protein (FMR1), Hrp59, insulin growth factor II mRNA-binding protein (IMP), Argonaute 2 (Ago2), Poly A Binding Protein (PABP), Heat shock cognate proteins 3 and 4 (Hsc70-3 and -4), several ribosomal proteins, and three proteins of unknown function, CG5787, CG14648, and CG5641 (Figure 1A and Table 1). Zn72D was previously shown to interact with eight proteins in a large-scale two-hybrid protein-protein interaction analysis [12]. Among these proteins, the only one identified as a Zn72D-interacting protein in our assay was CG5641, which has a 2'5'-oligoadenylate synthase motif and a DZF domain of unknown function also found in Zn72D. We confirmed that CG5641 and two additional proteins, FMR1 and Bel, co-IP with HA-Zn72D by western blotting (Figure 1B and 1C). Zn72D and four of the Zn72D-interacting proteins, Bel, FMR1, CG5641, and Hrp59, display a common pattern of localization during stages 13–16 of embryonic development [13-15], suggesting the possibility that the interaction of these proteins may play a role in regulation of central nervous system development.

**Table 1:** Identification of Zn72D-interacting proteins by mass spectrometry

**Annotation Symbol**	**Name**	**Number of Peptides**	**% Coverage**
CG7349	Argonaute 2	6	7
CG5215	HA-Zn72D	44	43
CG5787	CG5787	6	5
CG9748	Belle	4	5
CG6203	FMR1	2	3
CG4147	Hsc70-3	28	41
CG4264	Hsc70-4	16	26
CG14648	CG14648	8	14
CG9393	Hrp59	2	3
CG5119	PABP	11	25
CG1691	IMP	2	3
CG5641	CG5641	21	49
CG8280	EF1α48D	6	12
CG5641	CG5641	25	55
CG5502	RpL4	3	6
CG7434	RpL22	3	16
CG6779	RpS3	7	23
CG2168	RpS3A	6	20
CG17489	RpL5	4	18
CG11276	RpS4	18	64
CG1263	RpL8	4	17
CG10944	RpS6	3	10

While Zn72D is implicated regulating splicing of *mle *mRNA [10], the only Zn72D-interacting protein with a known role in splicing is Hrp59 [16,17]. Hrp59 depletion did not phenocopy Zn72D knockdown (data not shown), suggesting that Zn72D is unlikely to regulate *mle *splicing solely through Hrp59. The majority of the Zn72D-interacting proteins are involved or implicated in other aspects of RNA metabolism, including transport/nucleocytoplasmic shuttling of RNAs, RNA binding, RNA localization, translation, and RNA interference [18,11-26]. These data suggest that the *mle *mRNA splicing regulation by Zn72D may be indirect.

### Simultaneous knockdown of *Zn72D *and *bel *restored MSL complex localization to the X chromosome and X-linked gene expression

**Figure 2:**
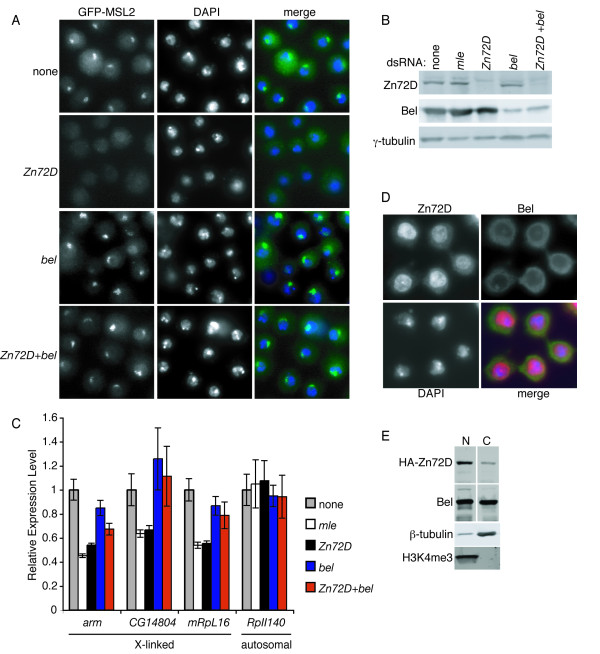
**Co-knockdown of *Zn72D *and *bel *restores MSL localization and X-linked gene expression**. (A) GFP-MSL2 expressing S2 cells were either untreated or treated with *Zn72D, bel*, or *Zn72D+bel *dsRNAs. The left panels show GFP fluorescence (green), the middle panels show DAPI staining to highlight the nucleus (blue), and right panels show the merged image. (B) Western blots with anti-Zn72D (top), anti-Bel (middle), or anti-γ-tubulin (bottom) as a loading control, in knockdowns indicated above. (C) qRT-PCR for the X-linked genes *arm*, *CG14804*, and *mRpL16 *and the autosomal gene *RpII140 *was done after S2 cells were untreated (gray bars), or treated with dsRNAs to knockdown *mle *(white), *Zn72D *(black), *bel *(blue), or *Zn72D+bel *(red). Samples were normalized first to *rp49 *and then to the untreated sample, setting untreated to 1. The error bars represent the average of three independent experiments, with qPCR performed in triplicate, and error was determined using standard error propagation methods. (D) Zn72D and Bel are both present in the cytoplasm. Cells were stained with antibodies to HA (red), Bel (green), and DAPI (blue) to delineate the nucleus. The nucleolar staining in the anti-Bel image was the result of antibody cross-reactivity, as when *bel *is knocked down, the nucleolar staining remained while the Bel staining decreased (data not shown). (E) Zn72D and Bel are present in both nuclear and cytoplasmic extracts. The cytoplasmic extract is enriched for β-tubulin and depleted for H3K4me3, whereas the nuclear extract is depleted for β-tubulin and enriched for H3K4me3.

We previously showed that Zn72D is necessary for generating productively spliced *mle *mRNA and assembly of the MSL complex on the X chromosome [10]. We tested whether knockdown of any protein that co-IPed with Zn72D would phenocopy *Zn72D *knockdown. None of these proteins were necessary for MSL complex localization, as assayed by accumulation of GFP-MSL2 on the X chromosome in S2 cells (Figure 2A and data not shown). Depletion of *pAbp*, *RpL4, RpS3, RpS3A, RpS4*, and *RpS6 *resulted in cell death, consistent with an essential role for these proteins. *Argonaute 2 *knockdown caused upregulation of GFP-MSL2, as did knockdown of another protein that regulates RNAi, Dicer-2 (data not shown). Upregulation of endogenous MSL2 was not observed when either *Argonaute 2 *or *Dicer-2 *was knocked down (data not shown), suggesting that upregulation of GFP-MSL2 was due to disruption of the Argonaute 2/Dicer-2 RNAi pathway. We also knocked down each protein that co-IPed with Zn72D in combination with *Zn72D *knockdown. We found that for one co-knockdown, *Zn72D *and *bel*, MSL complex localization to the X chromosome was restored (Figure 2A). *Zn72D *knockdown was equally effective alone or in combination with *bel *knockdown, as assayed by western blotting (Figure 2B). This suggests that knockdown of *bel *in cells depleted of Zn72D allows for production of sufficient MLE protein for assembly of the MSL complex.

Zn72D is required for proper X-linked gene expression in males [10]. We asked whether, in addition to restoring MSL complex localization to the X chromosome, combined knockdown of *Zn72D *and *Bel *returned X-linked gene expression to normal. S2 cells were either untreated or treated with dsRNAs against *mle*, *Zn72D*, *bel*, or *Zn72D*+*bel*, and levels of three X-linked transcripts and one control autosomal transcript were assayed by quantitative reverse transcription PCR (qRT-PCR). Expression of the autosomal gene *RpII140 *was unaffected by any of the knockdowns. The levels of the X-linked transcripts *arm*, *CG14804*, and *mRpL16 *were decreased by approximately twofold upon knockdown of *mle *or *Zn72D *(Figure 2C), consistent with a defect in dosage compensation. Knockdown of *bel *had little to no affect on X-linked gene expression (Figure 2C). Co-knockdown of *Zn72D *and *bel *restored X-linked gene expression of *CG14804 *and *RpL16 *and partially restored expression of *arm *(Figure 2C). Therefore, in addition to restoring MSL complex localization to the X chromosome, depletion of both *Zn72D *and *bel *either completely or partially restored proper X-linked gene expression.

Since our data suggest that Bel and Zn72D physically interact and both function in regulation of *mle *expression, we examined whether these proteins co-localize. While Zn72D is predominantly nuclear, it can also be detected in the cytoplasm, and Bel is detected in both the nucleus and cytoplasm (Figure 2D and 2E). Thus, Zn72D and Bel may interact in the nucleus, cytoplasm, or both.

Since both Bel, a DEAD box helicase, and Zn72D are potential RNA binding proteins, we examined whether their interaction was RNA-dependent. We assayed co-IP of these proteins in the presence or absence of RNase A (Figure 1C). Bel and HA-Zn72D co-IPed whether or not RNase A was added to the extract, indicating that the interaction between Zn72D and Bel is independent of RNA. More HA-Zn72D was immunoprecipitated from RNase A treated extracts than from untreated extracts, suggesting that a fraction of Zn72D may be present in RNA containing complexes and released upon RNase A treatment. Because these co-immunoprecipitations are not quantitative, it is possible that a fraction of the Zn72D that interacts with Bel is in an RNA containing complex.

### Co-knockdown of *Zn72D *and *bel *partially rescues *mle *mRNA splicing

**Figure 3:**
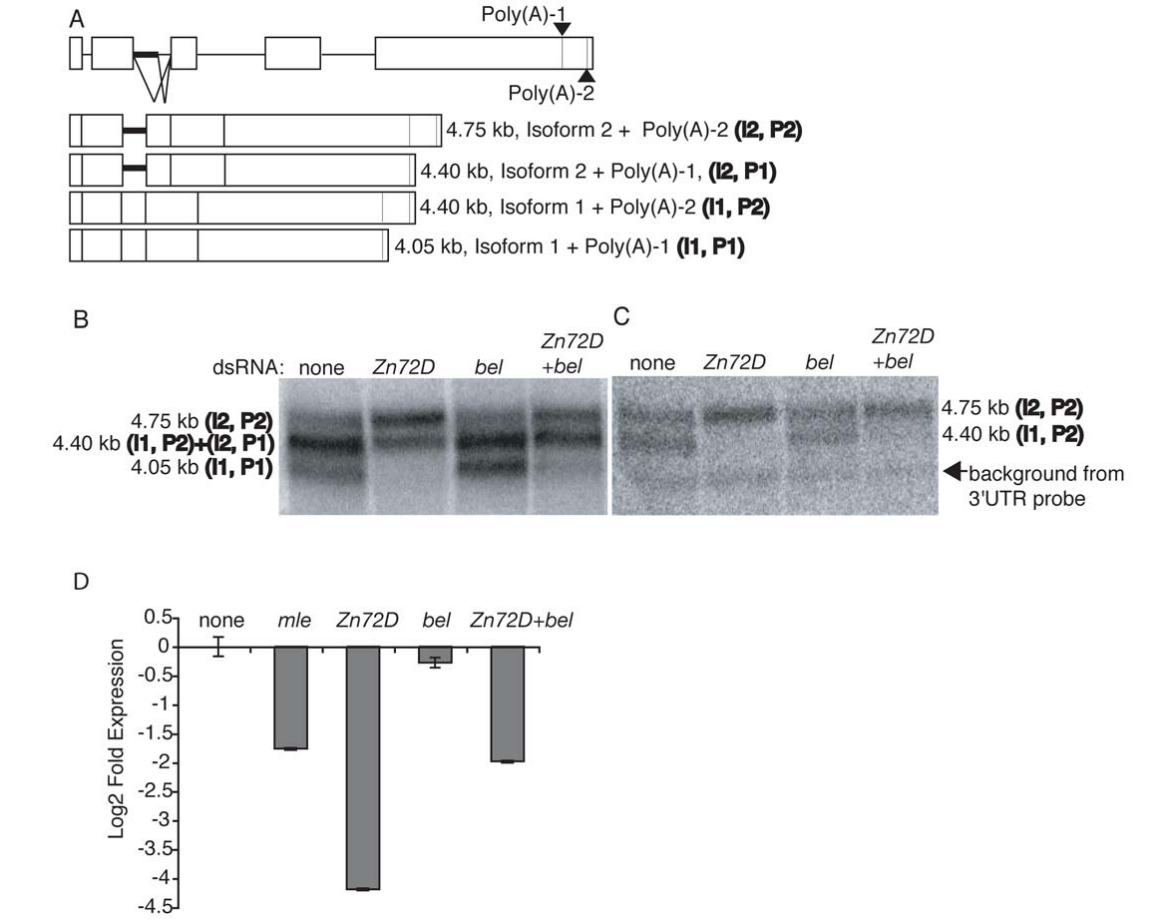
**Co-knockdown of *Zn72D *and *bel *partially restores productively spliced *mle *mRNA levels**. (A) Depicted is the *mle *gene, which contains 5 exons (white boxes) and 4 introns (black horizontal lines). Poly(A)-1 and -2 are depicted as gray vertical lines within the fifth exon. (Bottom) Four *mle *transcript isoforms differ in retention of part of intron 2 (Isoforms I1 and I2) and usage of the upstream or downstream poly(A) sites (P1 and P2). The partially retained intron is shown as a thicker horizontal black line. (B) Northern analysis with RNA collected from S2 cells untreated or treated with dsRNAs to *Zn72D*, *bel*, or *Zn72D+bel*. The blot was probed with a probe antisense to *mle *cDNA. The top band corresponds to isoform I2, P2; the middle band corresponds to both isoforms I1, P2 and I2, P1; and lower band corresponds to isoform I1, P1. In the absence of *Zn72D*, only I2 isoforms remain. Upon *Zn72D*+*bel *knockdown, there are predominantly I2 isoforms but a subtle increase in I1 isoforms compared to *Zn72D *knockdown. (C) The blot on the left was stripped and reprobed with a probe located between the two poly(A) sites. This probe only recognizes the P2 isoforms. There are similar slight increases in the level of correctly spliced I1 isoforms when comparing the left and right blots, indicating no difference in recovery of the two isoforms of *mle *mRNA that use alternative poly(A) sites. (D) S2 cells were untreated or treated with *mle*, *Zn72D*, *bel*, or *Zn72D+bel *dsRNAs and qRT-PCR was performed with primers to the *mle *transcript that specifically amplify isoform 1. Samples were normalized first to *rp49 *and then to the untreated sample, setting untreated to 1. The error bars represent the average of four independent experiments, with qPCR performed in triplicate, and error was determined using standard error propagation methods.

We next examined whether depletion of *bel *rescues the *mle *mRNA splicing defect that occurs upon *Zn72D *knockdown. Northern blot analysis of wild-type whole cell RNA with a cDNA probe showed three bands, which represent the two spliced forms (I1 and I2, with I1 indicating the productively spliced transcripts and I2 indicating the aberrantly spliced transcripts) combined with two polyadenylation sites (P1 and P2) (Figure 3A and 3B). Because the two splice sites are approximately the same distance apart as the two polyadenylation sites, the middle band represents a mixture of two isoforms (I1, P2 and I2, P1), while the upper and lower bands each represent a single isoform (I2, P2 and I1, P1 respectively). The *Zn72D *knockdown cells showed a considerable decrease in the intensity of the lower and middle bands (Figure 3B). Northern blot analysis using a 3'UTR probe, which detects only the P2 isoforms, showed that the decrease in intensity of the middle band was due to altered spliced site usage and not altered polyadenylation (Figure 3C). qRT-PCR, employing a primer set that specifically amplifies productively spliced *mle *transcripts [10], showed a 16-fold decrease in correctly spliced *mle *mRNA in *Zn72D *knockdown cells relative to wild-type cells (Figure 3D). Thus, there is a substantial decrease in levels of productively spliced *mle *transcripts upon *Zn72D *depletion, as previously reported [10].

Cells depleted for *bel *showed a similar distribution of *mle *mRNA isoforms as wild type cells (Figure 3B and 3C). Levels of productively spliced *mle *mRNA were slightly decreased upon knockdown of *bel *(Figure 3D). Thus *bel *knockdown alone does not significantly affect production of productively spliced *mle *mRNA. The combined knockdown of *Zn72D*+*bel *showed a four-fold decrease in the abundance of productively spliced *mle *mRNA isoforms (Figure 3B and 3C), a decrease that was not as great as that observed upon depletion of Zn72D alone (Fig. 3D). A similar result was observed when *Zn72D *and *bel *were knocked down in the female *Drosophila *Kc cell line, indicating that the effect on spliced *mle *transcript levels was not the result of improper dosage compensation in males (data not shown). Thus, there is some recovery of splicing of the *mle *transcript when *Zn72D *and *bel *are co-depleted, compared to knockdown of *Zn72D *alone, suggesting that *bel *may inhibit productive splicing of *mle *mRNA. However, *bel *knockdown alone does not increase the amount of productively spliced *mle *mRNA, indicating that *bel *depletion must be combined with *Zn72D *depletion for *bel *depletion to effect *mle *splicing. These data suggest that Bel and Zn72D may function together in regulation of *mle *mRNA processing.

### Zn72D and Bel regulate MLE protein levels

**Figure 4:**
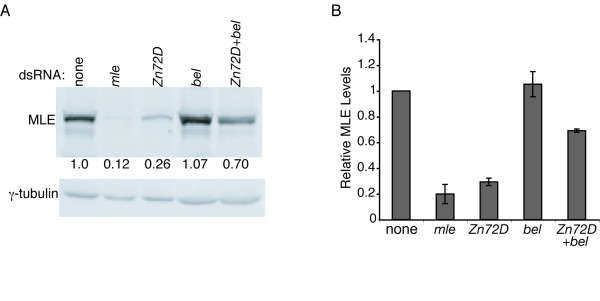
**Co-knockdown of *Zn72D *and *bel *restored MLE protein levels to 70% of normal levels**. (A) One representative quantitative western blot for MLE protein upon knockdown of *mle*, *zn72d*, *bel*, or *zn72d*+*bel*. (B) MLE levels were quantitated by first normalizing to the amount of γ-tubulin and then averaging the numbers obtained from the 3 experiments with 2 technical replicates per experiment. Standard deviation was found for the average of each experiment.

Since co-knockdown of *Zn72D *and *bel *resulted in restoration of MSL complex localization to the X chromosome, we expected that more MLE protein would be produced than when only *Zn72D *was knocked down. Quantitative western blots for MLE protein were performed on S2 cell extracts in which *mle*, *Zn72D*, *bel *or *Zn72D*+*bel *had been knocked down (Figure 4A and 4B). In the absence of *Zn72D*, the level of MLE protein decreased. Upon knockdown of *bel*, MLE protein levels were unaffected. When *Zn72D *and *bel *were knocked down together, MLE protein levels were restored to 70% of wild-type levels. This 70% of MLE protein is produced from 4-fold less productively spliced *mle *mRNA than is present in wild-type cells.

As high levels of protein are produced from a comparably small amount of *mle *transcript in the absence of *Zn72D *and *bel*, this suggests that perhaps the *mle *transcript is translated more efficiently or that MLE protein stability is increased in double knockdown cells. MLE protein levels did not change appreciably after treating cells for 8 hours with cycloheximide, suggesting that MLE is not an unstable protein (Additional File 1). In addition, MLE half-life was not affected by co-knockdown of *Zn72D *and *bel*, providing further evidence that the combined knockdown of *Zn72D *and *bel *does not affect the abundance of MLE protein by increasing its stability (data not shown). Therefore, these results suggest that co-knockdown of *Zn72D *and *bel *may release *mle *mRNA from translational inhibition, such that high levels of MLE protein are produced in spite of the decreased levels of productively spliced *mle *transcripts. This effect on translation could be through direct translational regulation or through regulation of cytoplasmic localization of *mle *transcripts, thus influencing translation efficiency.

The combined knockdown of *Zn72D *and *bel *resulted in increased levels of MLE protein from decreased amounts of productively spliced *mle *mRNA. Knockdown of *bel *alone did not have an appreciable affect on *mle *expression at either the mRNA or protein level. Together these data suggest that Bel may function, via its interaction with Zn72D, to regulate translation or transport of *mle *mRNA. There is considerable evidence implicating Bel in translational regulation: 1) Bel colocalizes at the oocyte posterior with the translational regulator and DEAD box helicase Vasa, and Vasa is required for the proper localization of Bel, thus implicating Bel as having a role in regulating local translation [11]. 2) *bel *knockdown in S2 cells reduced the amount of β-gal produced from an inducible LacZ transgene, without altering the abundance of LacZ mRNA [27]. 3) Bel is homologous to and can functionally substitute for the DEAD box helicase Ded1p protein in yeast, which is required for translation [28,11]. Biochemical and genetic evidence suggest that Ded1p plays a role, not only in translation, but also in splicing [29-31]. 4) Like Ded1p, the human Bel homologue, the DEAD box helicase DDX3, is a translational regulator that is also associated with the spliceosome [27,32-34]. DDX3 acts either as a translational repressor or activator, depending on the target mRNA [35,27,33]. These data have led to a model in which DDX3/Ded1p is loaded onto mRNA in the nucleus and then functions in translation in the cytoplasm [36]. This is particularly interesting, since Bel interacts with Zn72D, which regulates *mle *mRNA splicing. By associating with factors that regulate splicing, DDX3 and Bel may be recruited to specific transcripts to regulate their translation.

In addition to its role as a translational regulator, DDX3 is also implicated as a factor important for mRNA export and in localizing mRNAs for translation [37,35,39]. Recently, it was demonstrated that knockdown of *bel *does not affect nuclear export of bulk mRNA [27]. We found that Bel does not regulate nuclear export of *mle *transcripts, as we found that *mle *transcripts accumulated in the cytoplasm when *bel *was knocked down and when *Zn72D *and *bel *were knocked down together (Additional File 2). However, it is possible that Bel affects localization of *mle *mRNA within the cytoplasm to regulate translation. This would be consistent with Bel being specifically localized with Vasa at the posterior of the oocyte, where transcripts are deposited for localized translation.

The aforementioned data implicate Bel and its homologues in regulating gene expression at the level of mRNA localization and translation. Knockdown of *bel *by itself had no significant effect on MLE protein levels (Figure 4). Only when Bel depletion was combined with Zn72D depletion was an effect on MLE protein evident. This suggests two possibilities. First, that Bel is a weak suppressor of *mle *mRNA translation (either directly or via regulating cytoplasmic localization), such that when *mle *mRNA levels are high, Bel's effect on MLE protein levels is not significant. Alternatively, it may be that Zn72D blocks Bel activity on *mle *transcripts, and that these two proteins work together to finely tune production of MLE protein.

Why is it important to regulate MLE protein levels? MLE localizes to all chromosomes and throughout the nucleus when overexpressed [40,10]. Incorrect MLE expression is detrimental to the development of the fly, because heat shock over-expression of transgenic MLE protein results in male and female lethality [40]. It is possible that translational repression by Zn72D and Bel is one mechanism by which levels of MLE protein are tightly controlled. Expression of a transgenic *mle *cDNA in S2 cells resulted in overproduction of MLE protein; however, inclusion of the first two introns in the same transgene reduced the amount of MLE protein produced from the transgene [10]. This suggests that perhaps recruitment of Zn72D to the *mle *transcript has the effect of not only productively splicing the transcript but also targeting it for translational regulation.

Like Zn72D, its human homologue ZFR is also found mainly in the nucleus, with a subset in the cytoplasm. Cytoplasmic ZFR colocalizes in neuronal granules with Staufen2, a protein involved in mRNA transport and localization. ZFR interacts with and is required for cytoplasmic localization of the Staufen2^62 ^isoform [41]. As neuronal granules are involved in translational regulation and localization of mRNAs, these data suggest that ZFR may have a role regulating cytoplasmic localization of mRNAs. If Zn72D has a similar function in flies, it has the potential to regulate gene expression at two steps. Zn72D may first promote productive splicing of mRNAs and then later affect their cytoplasmic localization, which in turn may impact translation.

## Conclusion

We identified several proteins that interact with Zn72D, including the DEAD box helicase Bel. Co-knockdown of both *bel *and *Zn72D *restores the MSL complex localization to the X chromosome and dosage compensation of X-linked genes that was lost in the absence of Zn72D. In addition, we found that co-knockdown of *Zn72D *and *bel *resulted in restoration of MLE protein levels to 70% of wild-type levels, despite a four-fold reduction in properly spliced *mle *mRNA. These data implicate Zn72D and *bel *as being factors that target spliced mRNAs for localized, regulated translation in the cytoplasm.

## Methods

### Cell Culture and Generation of Stable Cell Lines

S2 cells were grown in Schneider's media plus 10% fetal bovine serum, penicillin and streptomycin. Cells were maintained according to the Invitrogen *Drosophila *Expression System Protocol. S2 cell lines expressing GFP-MSL2 and HA-Zn72D were described previously [10]. pAM-CG5641 (Myc-CG5641) was cloned using the Invitrogen Gateway system. 20 μg of the plasmid plus 1 μg pCoBlast (Invitrogen) was transfected into S2 cells using the protocol described in the Invitrogen *Drosophila *Expression System Protocol and selected with 15 μg/mL Blasticidin S HCl.

### Co-immunoprecipitation

S2 cells from a 10 cm dish were washed with 1×PBS and lysed in lysis buffer (50 mM Tris pH 7.4, 150 mM NaCl, 1 mM EDTA, 0.1% TritonX-100, supplemented with protease inhibitors and RNasin [Promega]). For Co-IPs that were performed in the presence of RNase A, RNAsin was left out of the buffer and RNAse A was added at a concentration of 0.1 mg/mL after sonication. The lysate was sonicated three times, 10 seconds each (on ice for one minute in between pulses) on setting 3, constant duty cycle on a Branson sonicator. The extract was clarified by centrifugation at 14,000 rpm for 15 min. at 4 degrees C. At least 600 ug extract was added to the 10 μL of Dynal Dynabeads (Invitrogen), precaptured with the appropriate antibody. Beads and extracts were rotated overnight at 4 degrees C and then washed 3 times with lysis buffer. Proteins were boiled off the beads in 1× sample buffer. (2× sample buffer: 8.3% glycerol, 1.25% SDS, 0.1 M Tris-HCl pH 6.7, 0.083 mg/mL bromophenol blue, 50 μL/mL 2-mercaptoethanol.) For large scale IPs for mass spectrometry, 45 mg of clarified S2 cell extract was added to 250 μL protein G Dynal Dynabeads preincubated with 25 μL HA.11 antibody (Covance). The proteins boiled off the beads were loaded on a 7.8% SDS-PAGE gel and which was later stained with G-250 coomassie blue.

### Cellular fractionation

Nuclear and cytoplasmic extracts were prepared as described in [42] (nuclear) and in [43] (cytoplasmic).

### On-line Capillary LC-MS and LC-MS-MS Analysis

Affinity-purified Zn72D-containing samples were separated by SDS-PAGE gel, in-gel digested and analyzed by LC-MS and LC-MS-MS as described previously [44]. Briefly, 1 μl aliquot of the digestion mixture was injected into an Ultimate capillary LC system via a FAMOS Autosampler (LC Packings, Sunnyvale, CA), and separated by a 75 μm × 15 cm reverse-phase capillary column at a flow rate of ~330 nL/min. The HPLC eluent was connected directly to the micro-ion electrospray source of a QSTAR Pulsar QqTOF mass spectrometer (Applied Biosystem/MDS Sciex, Foster City, CA). Typical performance characteristics were > 8000 resolution with 30 ppm mass measurement accuracy in both MS and CID mode. LC-MS data were acquired in an information-dependent acquisition mode, cycling between 1-s MS acquisition followed by 3-s low energy CID data acquisition. The centroided peak lists of the CID spectra were searched against the National Center for Biotechnology Information (NCBI) *Drosophila melanogaster *protein database using Batch-Tag, a program in the in-house version of the University of California San Francisco ProteinProspector package. The CID spectra were further inspected manually. Protein hits with more than two confident MSMS spectra are reported in the table.

### Quantitative western blotting

S2 cells +/- dsRNA treatment for 6 days were counted, spun down, and lysed in 1× Sample buffer (2× sample buffer: 8.3% glycerol, 1.25% SDS, 0.1 M Tris-HCl pH 6.7, 0.083 mg/mL bromophenol blue, 50 μL/mL 2-mercaptoethanol) at 5 × 10^4 ^cells/μL. Samples were boiled for 5 minutes and spun down at 14,000 rpm at 4 degrees C for 20 minutes. 15 μL lysate was loaded per lane of a 7.8% gel. Gels were transferred to nitrocellulose, blocked with 1% nonfat dry milk/0.05% Tween/1×PBS and probed overnight at 4 degrees with mouse anti-HA antibody at a 1:2000 dilution (HA.11, Covance), guinea pig anti-MLE antibody at 1:500 (gift from John Lucchesi), mouse anti γ-tubulin at 1:1000 (GTU-88, Sigma), mouse anti β-tubulin at 1:1000 (Developmental Studies Hybridoma Bank), rabbit anti-Bel at 1:2500 (gift from Paul Lasko), and chicken anti-Zn72D serum at 1:200 [10]. Donkey anti-guinea pig Cy3 (1:1000), anti-mouse Cy3 (1:500), anti-rabbit (1:1000), and anti-chicken (1:500) (Jackson ImmunoResearch), were used as a secondary antibodies, detected using a Typhoon 9400 instrument and quantitated using Quantity One software (Bio-Rad). MLE levels were normalized to γ-tubulin.

### RNAi

dsRNAs were added to S2 cells (~15 μg/mL) in 50% conditioned/50% fresh Schneider's media. The following primers were used to produce PCR products, which were used in T7 in-vitro transcription reactions to produce dsRNA:

*mle*(s): GGGCGGGTTTATGGCTTCGTACTCTAGCACC,

*mle*(as): GGGCGGGTAAGTTAAGCCAGTTGTCAACGC,

*bel(s)*: GGGCGGGTGTCTGGACTTGAATGGCGGC,

*bel*(as): GGGCGGGTCGTTGTAGTTGTCCTCGAAACGTC,

*Zn72D*(s): GGGCGGGTGTTGAACTTACAATCGCACAGC,

*Zn72D*(as): GGGCGGGTACTGGTATCAGCGTAAGATGGG,

*Zn72D*3'UTR(s): GGGCGGGTGCGGCGAGAATAGGTTATATAC,

*Zn72D*3'UTR(as): GGGCGGGTCCGCTTCGTTCTAGTATTTGTG.

### qRT-PCR

qRT-PCR on whole cell RNA was performed as described previously[10]. The primers used are described below:

*mle *QF1: CGGAACACGCTAGGAGCTTT, QR1: TGAGCGCCGGCACAT;

*rp49 *QF3: GCCGTAATTGTCGTTTTTGG, QR3: CGAACAGCGCACGGACTA;

*mRpL16 *QF2: TCAACACAGCCGGTCTTAAGTAT, QR2: GGCTGCTCCACATTCTGGTA; *arm*-F GCTGCTGAACGATGAGGATCA, *arm*-R: CCAAAGCGGCTACCATCTGA; *CG14804*-F: CTGAGCACAAGACGGCAGAG, *CG14804*-R:GAGGGTCACGTTCACCTTGC;

*RpII140*-F: CACAATGGCGGCGGTT, *RpII140*-R: ACGCAGATGTTCAGGCAGAGT [45].

### Northern blotting

Northern blots were performed with a NorthernMax Kit (Ambion) and BrightStar-Plus membrane (Ambion). 10 μg of RNA of whole cell extracts from S2 cells treated +/- Zn72D dsRNA for 6 days was loaded per lane. Blots were probed with a labeled DNA probe antisense to the full-length *mle *cDNA or to the 3'UTR of *mle *between the polyadenlyation sites, and exposed to a phosphor screen overnight.

## Authors' contributions

KAW and FC carried out the experiments. KW conceived the study and drafted the manuscript. BP participated in the study design and helped to draft the manuscript. All authors read and approved the final manuscript.

## Supplementary Material

Additional File 1 **Supplemental Fig. 1**. Over 8 hours of cycloheximide (chx) treatment, MLE protein levels do not increase. Click here for file

Additional File 2 **Supplemental Fig. 2**. *mle *mRNA is present in the cytoplasm when *bel *and *Zn72D+bel *are knocked down. Click here for file
