# Supplementary material for: The zinc finger protein Zn72D and DEAD box helicase Belle interact and control maleless mRNA and protein levels
Source: BMC Mol Biol. 2009 Apr 22;10:33. doi: 10.1186/1471-2199-10-33 (PMC2680859; doi:10.1186/1471-2199-10-33)
Supplement: Additional File 1 — Supplemental Fig. 1. Over 8 hours of cycloheximide (chx) treatment, MLE protein levels do not increase. [file 1471-2199-10-33-S1.pdf]

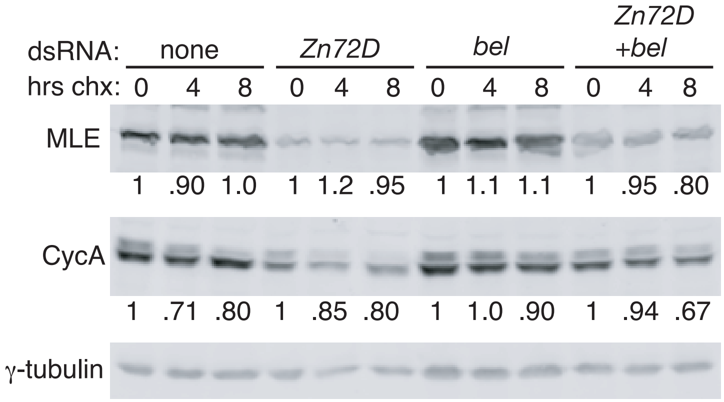

Supplemental Fig. 1. Over 8 hours of cycloheximide (chx) treatment, MLE protein levels do not increase. Over the same time period, CycA begins to turnover. Knockdown of Zn72D, bel, or Zn72D+bel does not affect MLE or CycA stability. CycA levels decreased over the time course even though we did not synchronize the cells, which leads to more obvious CycA turnover after chx treatment (Edgar et al. Distinct molecular mechanism regulate cell cycle timing at successive stages of Drosophila embryogenesis. Genes Dev. 8(4): 440-52. 1994). Zn72D knockdown lanes were underloaded on the CycA blot, resulting in lower overall levels of protein per lane, as judged by ponceS staining (data not shown). The stability over time for each knockdown was compared relative to the 0 time point and normalized to γ-tubulin.
