# Supplementary material for: The zinc finger protein Zn72D and DEAD box helicase Belle interact and control maleless mRNA and protein levels
Source: BMC Mol Biol. 2009 Apr 22;10:33. doi: 10.1186/1471-2199-10-33 (PMC2680859; doi:10.1186/1471-2199-10-33)
Supplement: Additional File 2 — Supplemental Fig. 2. mle mRNA is present in the cytoplasm when bel and Zn72D+bel are knocked down. [file 1471-2199-10-33-S2.pdf]

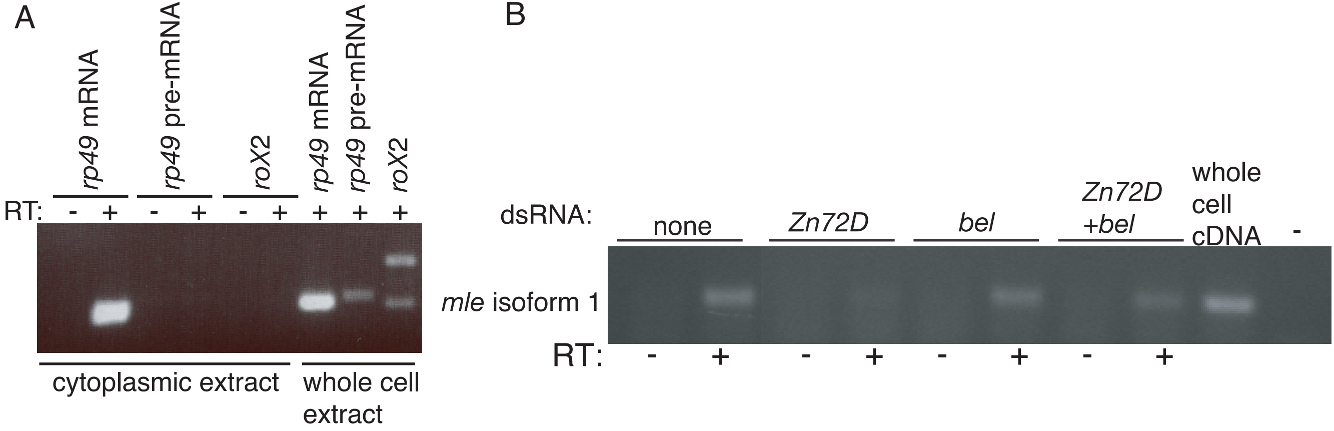

Supplemental Fig. 2. *mle* mRNA is present in the cytoplasm when *bel* and *Zn72D+bel* are knocked down. This suggests that Bel does not affect *mle* mRNA export. Cytoplasmic extracts were prepared as described in (A) Purity of cytoplasmic extracts was determined by PCR using primers that amplify *rp49* mRNA (cytoplasmic), *rp49* pre-mRNA (nuclear), and *roX2* (nuclear). (B) PCR using primers that recognize the correctly spliced *mle* mRNA indicate presence of *mle* mRNA in the cytoplasm in untreated, *Zn72D, bel,* and *Zn72D+bel* knockdowns. "-" indicates no cDNA added.
